# High prevalence of TB disease in contacts of adults with extrapulmonary TB

**DOI:** 10.1136/thoraxjnl-2017-210202

**Published:** 2017-11-16

**Authors:** Tom Wingfield, Peter MacPherson, Paul Cleary, L Peter Ormerod

**Affiliations:** 1 Department of Clinical Infection, Microbiology, and Immunology, Institute of Infection and Global Health, University of Liverpool, Liverpool, UK; 2 Department of Social Medicine, Karolinska Institutet, Stockholm, Sweden; 3 Department of Public Health, Cheshire and Merseyside Health Protection Team, Public Health England North West, Liverpool, UK; 4 Department of Clinical Sciences, Liverpool School of Tropical Medicine, Liverpool, UK; 5 Lancashire Postgraduate School of Medicine, University of Central Lancashire, Preston, UK

**Keywords:** clinical epidemiology, tuberculosis

## Abstract

UK guidelines no longer recommend routine screening of household contacts of adult patients with extrapulmonary TB (EPTB). From 27 March 2012 to 28 June 2016, we investigated the prevalence of active TB disease in household contacts of 1023 EPTB index cases in North West England, and compared estimates with: published new entrant migrant screening programme prevalence (~147/100 000 person-years); London-based contact screening data (700/100 000 contacts screened); and National Institute for Health and Care Excellence (NICE) new entrant TB screening thresholds (TB prevalence >40/100 000 people). Active TB disease prevalence in EPTB contacts was 440/100 000 contacts screened, similar to UK new entrant screening programmes, London EPTB contact prevalence and >10 times NICE’s threshold for new entrant screening. The decision to no longer recommend routine screening of EPTB contacts should be re-evaluated and cost-effectiveness analyses of screening strategies for EPTB contacts should be performed.

## Introduction

Formalised standardised contact tracing for TB has been recommended since 1983.^1^ The 2006 and 2011 National Institute for Health and Care Excellence (NICE) guidance^2 3^ recommend that ‘Screening should be offered to the household contacts of any person with active TB irrespective of the site of infection.’ This recommendation was based on five large contact tracing studies, which showed high yield of latent TB infection (LTBI) and active TB disease, even in contacts of patients with extrapulmonary TB (EPTB). However, in 2016, NICE no longer recommended routine contact tracing for household contacts of adults with EPTB.^4^ This decision has been questioned by the Joint TB Committee of the British Thoracic Society, and did not include published UK data from Edinburgh^5^ and Birmingham,^6^ which showed high rates of LTBI and active TB disease in EPTB contacts.

To examine the potential impact of the NICE 2016 recommendations on population TB case detection, we calculated the yield of active TB disease among household contacts of EPTB index cases, and compared this with recently published data from UK new-entrant migrant screening programmes and a descriptive study of contact screening in London.

## Methods

### Study design

A population-based retrospective study.

### Data source

Data were collected using Public Health England’s Enhanced TB Surveillance (ETS) system, supplemented with data from systematic multidisciplinary review during North West TB cohort audit.^7 8^

### Eligibility criteria

Participants were adult (≥18 years-old) residents of North West England notified to Public Health England ETS with microbiologically or clinically confirmed EPTB disease from 27 March 2012 to 28 June 2016, and who had ≥1 household contact, but were not part of a TB cluster (<25 contacts identified).

### Outcomes

#### Primary outcomes

The primary outcome was prevalence of active TB disease in contacts of eligible patients. Prevalence was calculated as the number of contacts identified to have active TB disease divided by the total number of contacts screened during the study period. Number needed to screen (NNS) to detect one case of active TB disease was derived from the prevalence. To adjust for potential impact of new entrant screening programmes, a sensitivity analysis was also performed that included only contacts of UK-born patients with EPTB.

#### Secondary outcomes

NNS and prevalence of LTBI and positive screening outcome (the sum of LTBI and active TB disease) in contacts of eligible patients were also estimated. Finally, we compared prevalence estimates from North West England with those obtained from UK new-entrant migrant screening programmes,^9^ recommended thresholds for screening of new entrants to the UK in NICE 2016 TB guidelines,^4^ and a recent descriptive study of contact tracing from the London area.^10^

### Statistical analysis

Proportions of contacts who had active TB disease, LTBI or a positive screening outcome in LTBI were summarised with CIs and compared between groups: UK-born versus non-UK-born long-term (above cohort median years since entry to UK) migrants versus non-UK-born recent (below cohort median years) migrants; and mediastinal lymph node or pleural TB versus other site of EPTB,^10^ using χ^2^ test for trend.

All statistical analyses were performed using Stata V.12 (StataCorp, College Station, Texas).

### Ethical approval

This study used anonymised routinely collected public health surveillance data and individual participant assent was not sought.

## Results

Between 27 March 2012 and 28 June 2016, up to 1026 (51%) index cases with EPTB met eligibility criteria, and had a total of 3652 household contacts screened (median 3; IQR 2–5).

**Figure 1:**
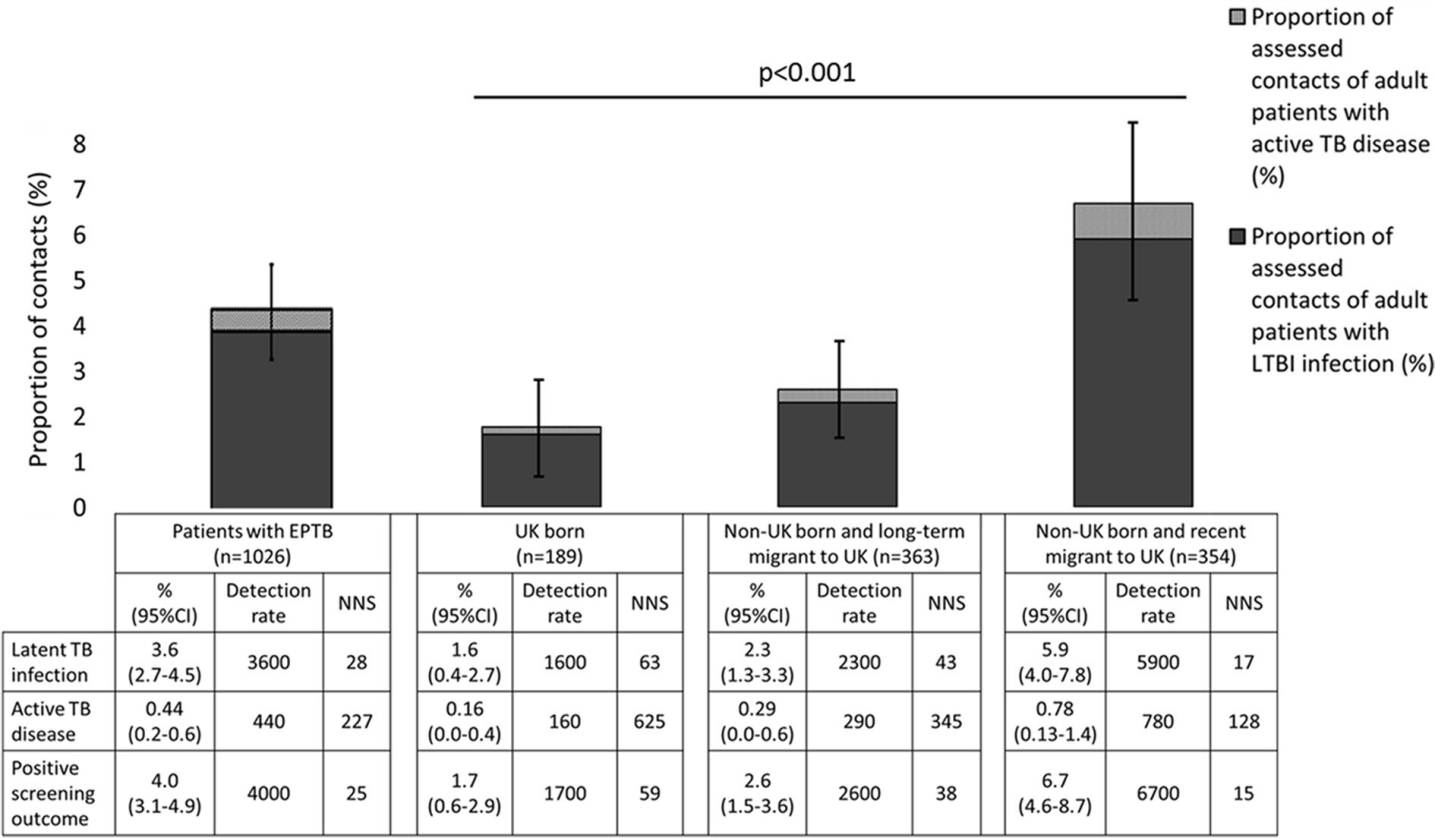
Prevalence of latent TB infection (LTBI), active TB disease and positive screening outcome in extrapulmonary TB (EPTB) contacts overall and by place of birth and entry to UK (n=1026). Detection rate is the number of cases of LTBI, active TB disease or positive screening outcome detected per 100 000 contacts screened. NNS is the number of patients needed to screen to detect one case of LTBI, active TB disease or a positive screening outcome in their screened contacts. Error bars represent 95% CIs and the P value is derived from χ^2^ test for trend comparing prevalence of LTBI, active TB disease and positive screening outcome in contacts of patients born in UK, born abroad with less recent entry and born abroad with recent entry (all P<0.001). Up to 946 of 1026 patients with EPTB had data available on country of birth and time in UK.

The prevalence of active TB disease, LTBI and positive screening outcome in EPTB contacts was 0.44% (95% CI 0.2 to 0.6), 3.6% (95% CI 2.7 to 4.5) and 4.0% (95% CI 3.1 to 4.9), respectively (figure 1). To detect one positive screening outcome, 25 contacts of EPTB cases would need to be screened (figure 1).

Eligible contacts of patients who were recent migrants to the UK (less than median duration of years since entry) compared with patients who were long-term migrants or UK born had higher prevalence of active TB disease (recent migrants 0.78% (0.13–1.4) vs long-term migrants 0.29% (0.0–0.6) vs UK born 0.16% (0.0–0.4), χ^2^ test for trend P<0.001, figure 1) and LTBI (5.9% (4.0–7.8) vs 2.3% (1.3–3.3) vs 1.6% (0.4–2.7), figure 1).

**Figure 2:**
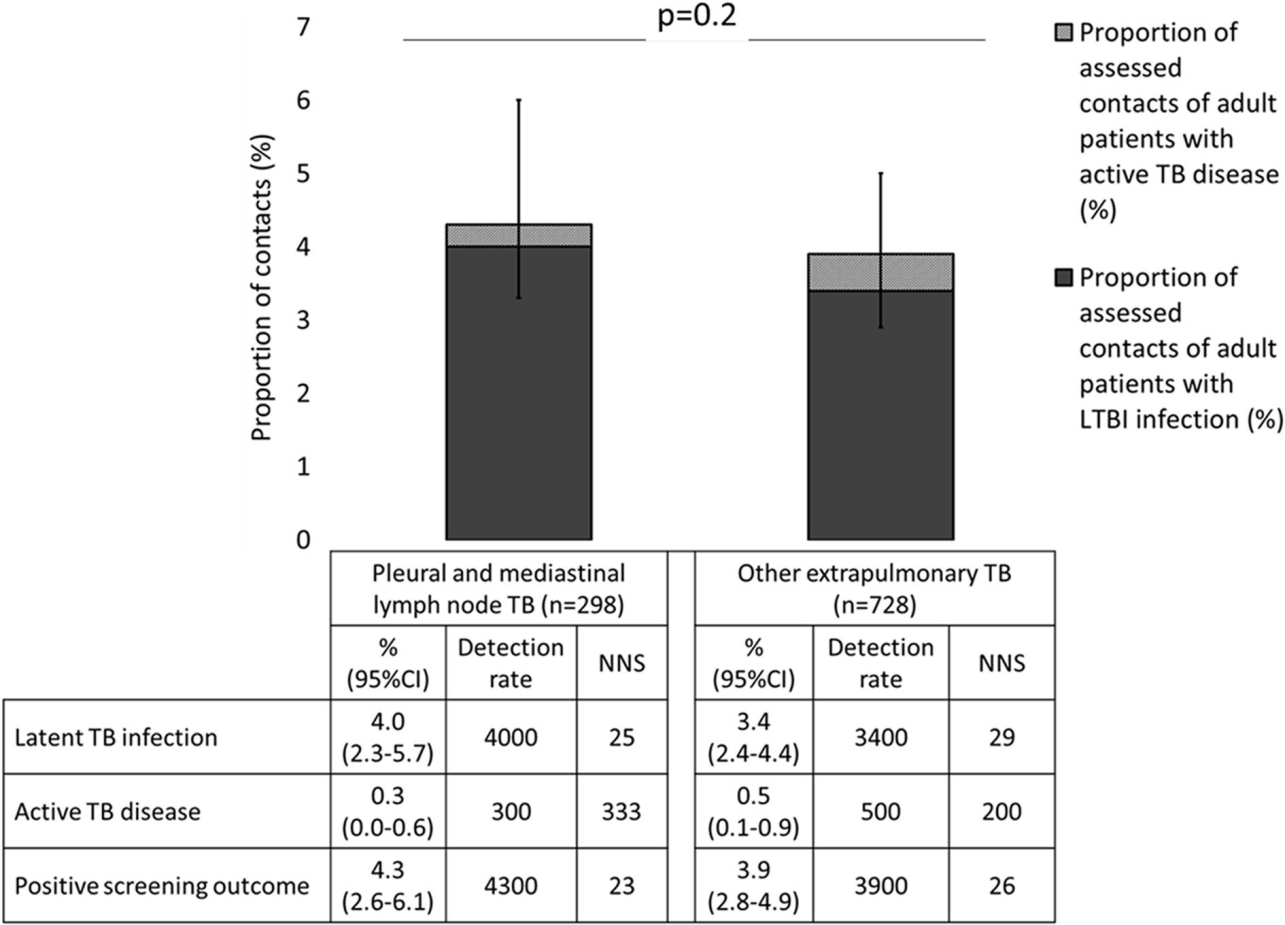
Prevalence of latent TB infection (LTBI), active TB disease and positive screening outcome in extrapulmonary TB (EPTB) contacts of patients with pleural or isolated mediastinal lymph node TB versus other sites of EPTB (n=1026). Detection rate is the number of cases of LTBI, active TB disease or positive screening outcome detected per 100 000 contacts screened. NNS is the number of patients needed to screen to detect one case of LTBI, active TB disease or a positive screening outcome in their screened contacts. Error bars represent 95% CIs and the P value is derived from χ^2^ test comparing prevalence of LTBI, active TB disease and positive screening outcome in contacts of patients with pleural or isolated mediastinal lymph node TB versus other sites of EPTB (P=0.2).

There was no significant difference between prevalence of active TB disease, LTBI or positive screening outcome in contacts of patients with pleural and isolated mediastinal lymphadenopathy versus contacts of patients with EPTB at other sites (figure 2).

## Discussion

The NICE 2016 TB guidelines no longer recommend routine screening for EPTB contacts, citing a lack of cost-effectiveness. However, our data from North West England, a low TB burden region, show that prevalence of active TB disease among all household contacts was high at 440 per 100 000, and was 167 per 100 000 when analysis was limited to contacts of UK-born EPTB cases. These data strongly suggest that screening of household contacts of EPTB cases could have substantial individual and public health benefits, and with recent data from London demonstrating similar findings, we urge that NICE guidance is reconsidered.

The active TB disease point prevalence we found is similar to a recent review of UK pre-entrant screening programmes (147 per 100 000 person-years),^9^ a London-based contact tracing study (700/100 000 contacts screened)^10^ and 10 times higher than that suggested by 2016 NICE guidelines as the country prevalence threshold for UK new entrant screening (>40 per 100 000 people per year).^4^

This study has some limitations. First, ETS data were collected retrospectively, meaning that despite regional data cleaning, some data may be missing or inaccurate. Second, despite complementary results to those from the London-based cohort,^10^ the North West population of TB-affected people may not be generalisable to the rest of England.

In conclusion, our findings show that the yield of active TB disease in EPTB contacts is substantially greater than the threshold advocated by NICE for new entrant screening and similar to that of a large London-based cohort. This suggests that, in England, the 2016 decision by NICE to no longer recommend routine EPTB contact tracing should be re-evaluated, including through studies of cost-effectiveness.
